# 230. Multicenter Assessment of the Accuracy of MIC Results for Colistin with MicroScan Dried Gram Negative MIC Panels using CLSI Breakpoints

**DOI:** 10.1093/ofid/ofac492.308

**Published:** 2022-12-15

**Authors:** Stefan Riedel, Maria Traczewski, Denise Beasley, Regina Brookman, Jose Diaz, Zabrina Lockett, Jennifer Chau

**Affiliations:** Beth Israel Deaconess Medical Center, Boston, Massachusetts; Clinical Microbiology Institute, Wilsonville, Oregon; Clinical Microbiology Institute, Wilsonville, Oregon; Beckman Coulter, Inc., West Sacramento, CA, California; Beckman Coulter, Inc., West Sacramento, CA, California; Beckman Coulter, Inc., West Sacramento, CA, California; Beckman Coulter, Inc., West Sacramento, CA, California

## Abstract

**Background:**

A multicenter study was performed to evaluate the accuracy of MIC results for colistin on a MicroScan Dried Gram Negative MIC (MSDGN) Panel when compared to results obtained with frozen broth microdilution panels prepared according to CLSI/EUCAST methodology. Note: Colistin is not available in all countries.

**Methods:**

MSDGN panels were evaluated at three clinical sites (including Beckman Coulter) by comparing MIC values obtained using the MSDGN panels to MICs obtained utilizing a CLSI/EUCAST broth microdilution reference panel. The study included 407 clinical isolates tested using the turbidity and Prompt® methods of inoculation. MSDGN panels were incubated in the WalkAway System (35 ± 1^°^C) and read on the WalkAway System, the autoSCAN-4 instrument, and read visually at 16-20 hours. Frozen reference panels were prepared and inoculated according to CLSI/EUCAST Joint Working Group Recommendations for MIC determination of Colistin (Polymyxin E) and incubated for 16-20 hours and read visually. CLSI M100 ED32 breakpoints (µg/mL) used for interpretation of MIC results were: Enterobacterales ≤ 2 I, ≥ 4 R; *P. aeruginosa* ≤ 2 I, ≥ 4 R.

**Results:**

Essential, categorical agreement, and categorical errors were calculated using MIC results from WalkAway reads for MSDGN panels in Table 1. All other read methods yielded similar results. Categorical agreement was not calculated for *Acinetobacter* spp. as MIC only is reported.

**Table 1:**
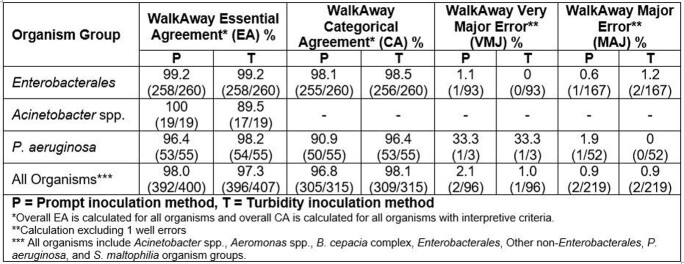
- Results

**Conclusion:**

This multicenter study showed that colistin MIC results for Gram Negative bacteria obtained with the MSDGN panel correlate well with MICs obtained using frozen reference panels with CLSI interpretive criteria.

© 2022 Beckman Coulter. All rights reserved. All other trademarks are the property of their respective owners.

Beckman Coulter, the stylized logo, and the Beckman Coulter product and service marks mentioned herein are trademarks or registered trademarks of Beckman Coulter, Inc. in the U.S. and other countries.

**Disclosures:**

**Stefan Riedel, M.D., PhD., D(ABMM), FCAP**, Beckman Coulter, Inc.: Clinical trial data collection funded by Beckman Coulter, Inc. **Maria Traczewski, BS**, Beckman Coulter, Inc.: Clinical trial data collection funded by Beckman Coulter, Inc. **Denise Beasley, BS**, Beckman Coulter, Inc.: Clinical trial data collection funded by Beckman Coulter, Inc. **Regina Brookman, BS**, Beckman Coulter, Inc.: Employee of Beckman Coulter **Jose Diaz, BS**, Beckman Coulter, Inc.: Employee of Beckman Coulter **Zabrina Lockett, PhD**, Beckman Coulter, Inc.: Employee of Beckman Coulter **Jennifer Chau, PhD**, Beckman Coulter, Inc.: Employee of Beckman Coulter.

